# Colorectal Cancer Screening Modalities in Chinese Population: Practice and Lessons in Pudong New Area of Shanghai, China

**DOI:** 10.3389/fonc.2019.00399

**Published:** 2019-06-04

**Authors:** Wei-miao Wu, Yingying Wang, Hui-ru Jiang, Chen Yang, Xiao-qiang Li, Bei Yan, Yi Zhou, Wang-hong Xu, Tao Lin

**Affiliations:** ^1^Fudan University School of Public Health, Key Lab of Health Technology Assessment, National Health Commission of the People's Republic of China (Fudan University), Shanghai, China; ^2^Fudan University Pudong Institute of Preventive Medicine, Pudong New Area, Shanghai, China; ^3^Center of Disease Prevention and Control in Pudong New Area of Shanghai, Shanghai, China

**Keywords:** colorectal cancer, screening, risk assessment, risk score, fecal immunochemical tests

## Abstract

**Background:** Parallel test of risk stratification and two-sample qualitative fecal immunochemical tests (FITs) are used to screen colorectal cancer (CRC) in Shanghai, China. This study was designed to identify an optimal initial screening modality based on available data.

**Methods:** A total of 538,278 eligible residents participated in the program during the period of January 2013 to June 2017. Incident CRC was collected through program reporting system and by record linkage with the Shanghai Cancer Registry up to December 2017. Logistic regression model was applied to identify significant factors to calculate risk score for CRC. Cutoff points of risk score were determined based on Youden index and defined specificity. Sensitivity, specificity, and positive predictive values (PPVs) were computed to evaluate validity of assumed screening modalities.

**Results:** A total of 446 CRC were screen-detected, and 777 interval or missed cases were identified through record linkage. The risk score system had an optimal cutoff point of 19 and performed better in detecting CRC and predicting long-term CRC risk than did the risk stratification. When using a cutoff point of 24, parallel test of risk score, and FIT were expected to avoid 56 interval CRCs with minimal decrease in PPV and increase in colonoscopy. However, the observed detection rates were much lower than those expected due to low compliance to colonoscopy.

**Conclusions:** Risk score is superior to risk stratification used in the program, particularly when combined with FIT. Compliance to colonoscopy should be improved to guarantee the effectiveness of CRC screening in the population.

## Introduction

Colorectal cancer (CRC) is one of the most common cancers globally, leading to over 1.8 million new cases and 881,000 deaths in 2018 (1). In China, CRC ranks second in incidence and fourth in death of all cancers (http://gco.iarc.fr/, access date: April 4, 2019). The rapid increasing incidence and mortality of the disease (2) and the proven effectiveness of screening in CRC prevention and control (3) motivate the Chinese government to perform and scale up population-based CRC screening around the country.

Population-based CRC screening has been implemented in many countries as a National Cancer Screening Program (4). Multiple methods were used in these programs, mainly stool-based tests like guaiac-based fecal occult blood test, fecal immunochemical test (FIT), and stool DNA testing, and direct visualization tests such as flexible sigmoidoscopy, colonoscopy, double-contrast barium enema, CT colonography, and video capsule colonoscopy (5). In resource-limited settings, serial use of risk assessment and FIT were conducted to improve cost-effectiveness of screening (6). In Jiashan County of Zhejiang Province of China, however, parallel use of a questionnaire-based risk assessment and two-sample qualitative FITs were conducted as an initial screening method to increase sensitivity of screening. It was reported that in Chinese population, the sensitivity and specificity of one positive qualitative FIT were 90.4 and 53.8%, respectively, for CRC, and those of two positive qualitative FITs were 80.8 and 75.1%, respectively (7). The pilot study in Jiashan County showed that the parallel test modality performed well in detecting early colorectal neoplasms, and the positive predictive value (PPV) reached 2.7% (8, 9).

Based on the evidence, the Shanghai government launched a pilot community-based CRC screening project in 2008. Three-year practice using a similar screening protocol of Jiashan County showed a great improvement in detection of early-stage CRC (10). In 2013, a large-scale screening program was launched as a major public health service project, making Shanghai one of the earliest cities in China to undertake mass screening of CRC. So far, three rounds of screening have been performed, and the results of the first round validated the effectiveness of parallel use of risk stratification and FIT (11). The screening modality with a high sensitivity, however, has led to a high false positive rate and thus low compliance to further colonoscopy examination (12, 13), limiting the effectiveness of screening.

In this study, we took advantage of the database developed in screening practice in Pudong New Area of Shanghai, China, to optimize the risk assessment tool and seek an optimal initial screening protocol for CRC in this population.

## Materials and Methods

### Study Participants

Almost all guidelines recommend CRC screening for asymptomatic individuals between ages of 50 and 75 years (5, 14, 15) as mortality benefit is greatest for patients aged 50–70 years. However, in Shanghai, one of the most aging cities in China, the service was also provided to residents aged 76–79 years old to achieve equity in health care (10). Therefore, the inclusion criteria were defined as follows: (1) permanent residents of Shanghai, (2) living in Pudong New Area of Shanghai, (3) aged 50–79 years, and (4) beneficiaries of the basic medical insurance of Shanghai.

The first round of screening was conducted in 2013, the second round covered 3 years from January 2014 to December 2016, and the third round was planned from January 2017 to December 2019. Through community mobilization, a total of 538,278 eligible volunteers attended initial screening of CRC during the period of January 1, 2013 to June 30, 2017 and were included in this analysis.

This study was approved by the Medical Ethics Committee of the Center for Disease Control and Prevention in Pudong New Area of Shanghai, China, and oral consent was obtained from each participant of the screening program.

### Screening Procedure

A two-stage sequential screening was designed and conducted in all 15 districts of Shanghai in 2013. A questionnaire-based risk assessment and two-sample qualitative FIT were used as initial screening.

### Risk Stratification

The participants were regarded as positive in risk assessment if they had one of the following events: (1) a history of any cancer; (2) a history of polyps; (3) a family history of CRC in a first-degree relative and/or at least two of the following events: (a) chronic coprostasis, (b) chronic diarrhea, (c) phlegmatically blood feces, (d) serious unhappy life events such as death among first-degree relatives, (e) chronic appendicitis or appendectomy, and (f) chronic cholecystitis or cholecystectomy.

### Fecal Immunochemical Test

Two stool samples were collected with an interval of 1 week by community healthcare staff and tested in a local hospital by contracted experienced technicians. Three different parts were taken from each stool sample and then mixed and washed by special buffer solution. Each sample was collected in a tube, including about 5 ml moist stool content. A qualitative FIT test was conducted in 5 min after collection using colloidal gold assay (monoclonal antibody), with a positivity threshold of 100 ng/ml of sample solution. FIT test kits were purchased from Shanghai Lijun Medical Co. Ltd., China.

### Colonoscopy

Individuals with a positive FIT test or a positive risk assessment were regarded as positive in the first stage and were invited to undergo a colonoscopy as the second stage of screening. Colonoscopies were required to be performed in one of the 13 designated hospitals, where polyps and adenomas were removed once diagnosed. The risk assessment and FITs were administered free to participants, but colonoscopy was paid by basic medical insurance of Shanghai.

### Data Collection

To evaluate the effectiveness of the CRC screening program, we took all subjects as members of a prospective cohort. A 12-digit barcode was assigned to each participant at recruitment to follow screening results. Baseline demographic information and risk factors were collected through in-person interview using a structured questionnaire. The barcode appeared on the fecal collect tube, and when participants returned the tube, the FIT results were entered into the reporting system by scanning the barcode. The results of colonoscopic and histopathologic examinations were entered using the same barcode in designated hospitals and submitted monthly by the local community healthcare staff to the Center for Disease Control and Prevention in Pudong New Area of Shanghai through an internet-based reporting system.

**Figure 1:**
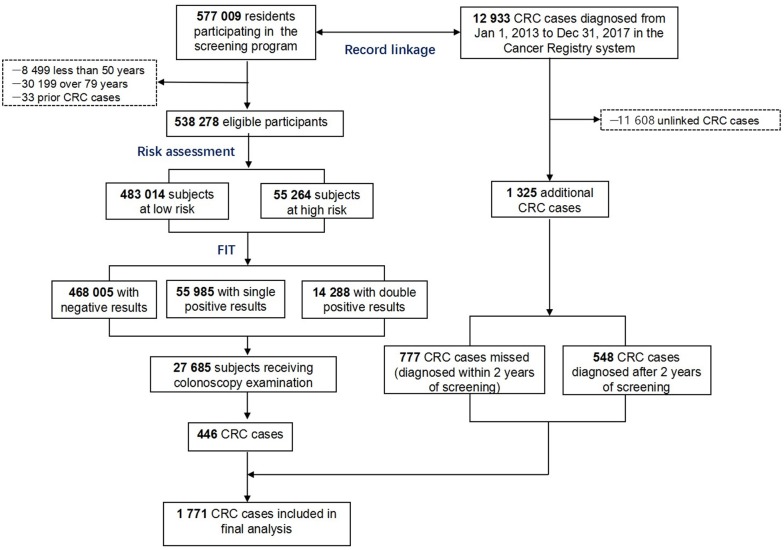
Flow chart for subject recruitment and data collection.

Newly diagnosed CRCs were obtained from the program reporting system as screen-detected cancers and supplemented by record linkage with the Shanghai Cancer Registry up to December 31, 2017 using unique ID numbers (Figure 1). Interval CRC was defined as those detected within 2 years after a negative initial screening test, while missed cases referred to those detected within 2 years after a positive initial screening test.

### Quality Control

The process of the screening program was supervised by the staff in the Center for Disease Control and Prevention in Pudong New Area of Shanghai who organized annual training for physicians, planned progress of the screening program, monitored screening tests, and supervised data collection and data entry. The final database was double-checked and verified to improve quality. Field quality control was conducted by community health care staff who were motivated by subsidies according to workload and quality assessment.

### Statistical Analysis

Positive rate was calculated as the number of subjects positive in the respective screening test divided by the number of all participants of the test. Observed detection rates were calculated as the number of screen-detected CRC divided by the number of all participants, while expected detection rates were calculated as the number of prevalent CRC (screen-detected, interval, and missed CRC) divided by the number of all participants.

Fisher exact test was used to test the differences in positive rates and detection rates. *Kappa* coefficients were used to evaluate consistency of stratified risk with FIT results. Logistic regression model for prevalent CRC cases was fitted by backward selection with age, sex, education, and risk factors listed in the questionnaire to identify significant factors to construct CRC risk score. Risk score was calculated by multiplying the β-coefficients of the significant variables by 10 and rounding to the nearest integer (16). Receiver operating characteristic (ROC) curve was obtained by plotting sensitivity against 1-specificity to evaluate performance of risk score and risk stratification used in the program. The optimal cutoff point of risk score was identified based on Youden index, which was at the maximum sum of the sensitivity and specificity-1 (16). The cutoff point at the same specificity of risk stratification was also used to compare PPVs of the two risk assessment methods.

In order to testify the stability of the present model, we developed a model in randomly selected 90% of the overall sample according to the above-mentioned analysis method and validated in the remaining 10% of the sample. The above progress was repeated 10 times. Significant risk factors in 10 subgroups were identical to those in the whole samples, and the areas under ROC curve (AUC) ranged from 0.644 to 0.664 for risk score. Sensitivity, specificity, and PPV were computed to evaluate validity of assumed screening modalities.

Person-years of observation was used to calculate overall incidence [95% confidence intervals (CIs)] of CRC by subgroups. The period of observation was further split into two intervals (within 2 years and ≥2 years of screening) to calculate incidence (95% CI) of CRC during each period. Sensitivity analysis was performed by defining interval and missed CRCs as those detected within 3 years after an initial screening test.

All statistical analysis was performed in the Statistics Analysis System version 9.4 (SAS 9.4).

## Results

### Demographic Characteristics of the Participants

In the program, a total of 538,278 residents participated in the screening program, accounting for 39.7% of all eligible residents (Table 1). More women and individuals aged 60–69 years participated in the program. Among all subjects, 55,264 (10.0%) were stratified as high-risk individuals, and 70,273 (13.1%) were positive in at least one FIT. As a result, a total of 115,247 (21.0%) participants positive in risk assessment or in FIT were considered as positive in the initial screening test and were advised to have a further colonoscopy examination. The positive rate increased with age and was higher in men and in the residents with college education or higher (*p* < 0.0001). Of all positive subjects in initial screening tests, only 27,097 (23.5%) had a colonoscopy examination, whereas 588 negative subjects had colonoscopy for unknown reasons.

**Table 1:** Positive rates of screening tests and compliance to colonoscopy by baseline demographic characteristics of participants.

**Demographic characteristics**	**No. of residents**	**Participants of initial screening, *n* (%)**	**Risk assessment positive, *n* (%)**	**FIT positive, *n* (%)**	**Initial screening positive, *n* (%)**	**Attended colonoscopy, *n* (%)**
All subjects	1,356,068	538,278 (39.7)	55,264 (10.0)	70,273 (13.1)	115,247 (21.0)	27,685 (24.0)
Sex						
Men	663,664	219,698 (33.1)	20,943 (9.5)	32,232 (14.7)	48,660 (22.1)	12,473 (25.6)
Women	692,404	318,580 (46.0)	34,321 (10.8)	38,041 (11.9)	66,587 (20.9)	15,212 (22.8)
Age group (years)						
50–54	234,537	42,784 (18.2)	3,492 (8.2)	3,937 (9.2)	6,982 (16.3)	1,804 (25.8)
55–59	277,152	94,275 (34.0)	8,498 (9.0)	10,669 (11.3)	17,770 (18.8)	4,865 (27.4)
60–64	291,245	141,133 (48.5)	14,097 (10.0)	18,106 (12.8)	29,703 (21.0)	8,029 (27.0)
65–69	207,614	148,444 (71.5)	15,932 (10.7)	20,748 (14.0)	33,585 (22.6)	7,858 (23.4)
70–74	117,743	75,643 (64.2)	8,895 (11.8)	11,052 (14.6)	18,139 (24.0)	3,803 (21.0)
75–79	227,077	35,999 (15.9)	4,350 (12.1)	5,761 (16.0)	9,068 (25.2)	1,326 (14.6)
Education						
No formal education	–	24,777	2,255 (9.1)	3,196 (12.9)	5,013 (20.2)	1,286 (25.7)
Primary school	–	159,868	11,713 (7.3)	19,736 (12.3)	29,221 (18.3)	8,444 (28.9)
Middle or occupational school	–	313,951	34,580 (11.0)	41,470 (13.2)	69,792 (22.2)	16,093 (23.1)
College or above	–	39,682	6,716 (16.9)	5,871 (14.8)	11,221 (28.3)	1,862 (16.6)

### Comparison of Risk Stratification and Risk Score in Detecting Colorectal Cancer

Risk score developed in this study included age group (50–54 years: score 0; 55–59 years: score 6; 60–64 years: score 9; 65–69 years: score 11; 70–74 years: score 14; 75–79 years: score 16), sex (women: score 0; men: score 6), chronic diarrhea (never: score 0; ever: score 3), phlegmatically blood feces (never: score 0; ever: score 12), polyps (never: score 5; ever: score 0), serious unhappy life events (never: score 0; ever: score 3), and family history of CRC (never: score 0; ever: score 6).

**Figure 2:**
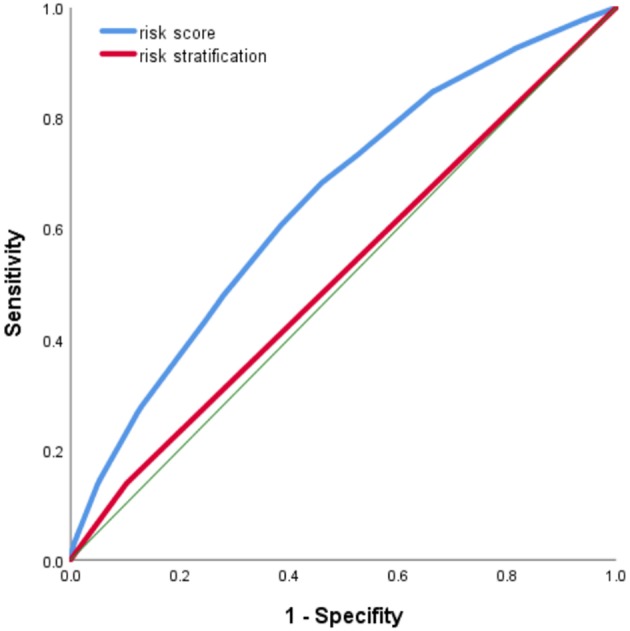
Receiver operating characteristic (ROC) curve of risk score in identifying colorectal cancer (CRC) cases.

The score ranged from 0 to 49, with an optimal cutoff point of 19. The cutoff point increased to 24 at the similar specificity of risk stratification used in the program (89.7%). The risk score performed better in detecting CRC than risk stratification, with AUC being 0.655 vs. 0.526 for risk stratification (Figure 2).

The factors for risk assessment were not well-consistent with FIT results, with an agreement ranging from 80.9 to 86.0% and a Kappa coefficient from 0.01 to 0.03 *(p* < 0.0001). The low agreement with FIT was also observed for overall risk assessment, with an agreement of 80.5% and a Kappa coefficient of 0.06 with risk stratification (*p* < 0.001), and an agreement of 54.7% and a Kappa coefficient of 0.04 with risk score (*p* < 0.001) (Table 2).

**Table 2:** Consistency in results of risk assessment and FIT in CRC screening.

**Risk assessment**	**FITs**		**Agreement**	**Kappa**	***P* value**
	**Any positive**	**Double negative**			
**Items for risk assessment**					
Chronic diarrhea					
Ever	4,261	20,715			
Never	66,012	447,290	83.9	0.02	< 0.0001
Chronic coprostasis					
Ever	5,497	26,617			
Never	64,776	441,388	83.0	0.03	< 0.0001
Phlegmatically blood feces					
Ever	2,077	7,320			
Never	68,196	460,685	86.0	0.02	< 0.0001
Chronic appendicitis/appendectomy					
Ever	6,598	35,749			
Never	63,675	432,256	81.5	0.02	< 0.0001
Cholecystitis or cholecystectomy					
Ever	6,903	39,362			
Never	63,370	428,643	80.9	0.02	< 0.0001
Serious unhappy life events					
Ever	1,373	6,481			
Never	68,900	461,524	86.0	0.01	< 0.0001
History of any cancer					
Ever	1,814	9,484			
Never	68,459	458,521	85.5	0.01	< 0.0001
Colon polyps					
Ever	2,375	8,736			
Never	67,898	459,269	85.8	0.02	< 0.0001
CRC in first degree relatives					
Positive	2,516	11,856			
Negative	67,757	456,149	85.2	0.02	< 0.0001
**Overall risk assessment**					
Risk stratification					
High risk	10,290	44,974			
Low risk	59,983	423,031	80.5	0.06	< 0.0001
Risk score					
≥19	37,065	210,749			
<19	33,208	257,256	54.7	0.00	< 0.0001

*CRC, colorectal cancer; FIT, fecal immunochemical test*.

### Detection Rates of Colorectal Lesions by Subgroups

A total of 446 CRC cases were screened and reported, and as many as 777 missed or interval cases were identified through record linkage with the Shanghai Cancer Registry possibly due to low compliance to colonoscopy. Detection rates, both observed and expected, were significantly higher in high-risk individuals defined by risk stratification, risk score, and FIT and were the highest (20.8/1,000 and 38.7/1,000, respectively) among subjects with high-risk score and positive double FIT. Detection rates of precancerous lesions (advanced adenoma, small tubular adenoma, serrated adenoma, villous adenoma, hamartoma, high- and low-grade dysplasia, tubular villous adenoma, etc.) were also higher in high-risk subjects defined by risk stratification, risk score, and FIT (Table 3).

**Table 3:** Detection rates of colorectal lesions by initial screening results.

**Methods of screening**	**No. of subjects**	**Attended colonoscopy, *n* (%)**	**Precancerous lesions**^**a**^		**CRC**			
			**No. of detected cases**	**Detection rate (1/1,000)**	**No. of detected CRC**	**Detection rate (1/1,000)**	**No. of prevalent CRC^**b**^**	**Expected detection rate (1/1,000)**
**Risk stratification**								
Low risk	483,014	18,356 (3.8)	2,537	5.3	366	0.8	1,035	2.1
High risk	55,264	9,329 (16.9)	908	16.4	80	1.4	188	3.4
**Risk score**								
<19	290,464	13,831 (4.8)	1,370	4.7	134	0.5	383	1.3
≥19	247,814	13,854 (5.6)	2,075	8.4	312	1.3	840	3.4
**FIT**								
Double negative	468,005	6,353 (1.4)	545	1.2	19	0.0	404	0.9
Single positive	55,985	16,250 (29.0)	2,034	36.3	191	3.4	384	6.9
Double positive	14,288	5,082 (35.6)	866	60.6	236	16.5	435	30.4
Any positive	70,273	21,332 (30.4)	2,900	41.3	427	6.1	819	11.7
**Risk stratification and FIT**								
Low risk and double FIT (–)	423,031	588 (0.1)	77	0.2	7^c^	–	350^c^	–
Single FIT positive only	47,953	13,583 (28.3)	1,726	36.0	163	3.4	324	6.8
Double FIT positive only	12,030	4,185 (34.8)	734	61.0	196	16.3	361	30.0
High risk only	44,974	5,765 (12.8)	468	10.4	12	0.3	54	1.2
High risk and single FIT (+)	8,032	2,667 (33.2)	308	38.3	28	3.5	60	7.5
High risk and double FIT (+)	2,258	897 (39.7)	132	58.5	40	17.7	74	32.8
**Risk score and FIT**								
Risk score < 19 and double FIT (–)	257,256	3,190 (1.2)	221	0.9	4^c^	–	129^c^	–
Single FIT positive only	27,192	8,343 (30.7)	827	30.4	66	2.4	139	5.1
Double FIT positive only	6,016	2,298 (38.2)	322	53.5	64	10.6	115	19.1
Risk score ≥19 only	210,749	3,163 (1.5)	324	1.5	15	0.1	275	1.3
Risk score ≥19 and single FIT (+)	28,793	7,907 (27.5)	1,207	41.9	125	4.3	245	8.5
Risk score ≥19	8,272	2,784 (33.7)	544	65.8	172	20.8	320	38.7
and double FIT (+)								

a Including advanced adenoma, small tubular adenoma, serrated adenoma, villous adenoma, hamartoma, high- and low-grade dysplasia, and tubular villous adenoma.

b Including screened CRC, missed CRC, and/or interval CRC diagnosed within 2 years after initial screening among positive subjects.

c *Potential interval CRC*.

**Figure 3:**
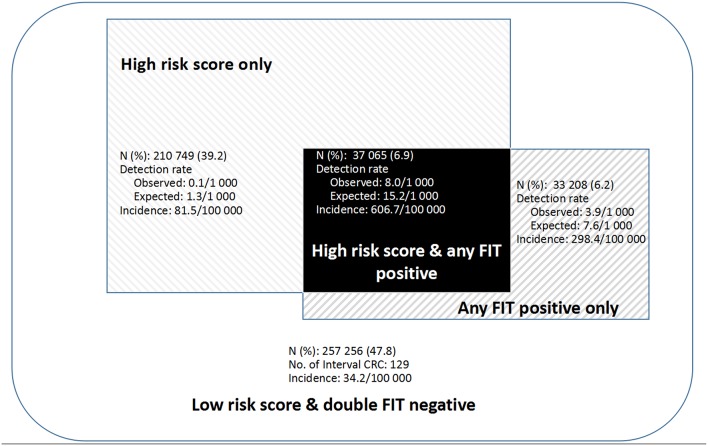
Performance of combined use of fecal immunochemical test (FIT) with risk score.

As shown in Figure 3, CRC incidence was 81.5/100,000 among subjects with high-risk score only, significantly higher than 34.2/100,000 among those with low-risk score and negative double FIT. Detection rates and incidence of CRC doubled among subjects with high-risk score and any FIT positive than in those with any FIT positive only.

### Incidence of Colorectal Cancer Along Follow-Up Time

As shown in Table 4, risk stratification, risk score, and FIT performed well in predicting CRC risk, with significant higher incidence of CRC after 2 or 3 years of initial screening in positive subjects. With the least number of interval CRC cases, parallel use of FIT and risk score performed better than modality used in the program in identifying individuals at high risk of CRC.

**Table 4:** Incidence and 95% CI of CRC by initial screening results.

**Screening methods**	**No. of subjects**	**No. of CRC cases**	**Incidence (95%CI) (1/100,000)**	**Incidence (95% CI)**		**Incidence (95% CI)**	
				**Within 2 years of screening**	**After 2 years of screening**	**Within 3 years of screening**	**After 3 years of screening**
**Risk stratification**							
Low risk	483,014	1,526	103.9 (98.8, 109.2)	52.5 (47.9, 57.6)	177.0 (166.7, 188.0)	54.5 (50.5, 58.9)	300.4 (281.2, 320.8)
High risk	55,264	245	146.4 (129.2, 165.9)	92.1 (74.9, 113.3)	222.4 (190.0, 260.4)	84.7 (70.4, 101.9)	388.6 (327.7, 460.9)
**Risk score**							
<19	290,464	563	64.1 (59.0, 69.6)	30.5 (26.1, 35.7)	112.4 (102.0, 124.0)	34.5 (30.5, 39.2)	183.8 (164.7, 205.1)
≥19	247,814	1,208	159.5 (150.7, 168.7)	87.1 (78.8, 96.2)	260.7 (243.5, 279.1)	84.6 (77.6, 92.3)	450.6 (418.4, 485.3)
**FIT**							
Negative	468,005	792	55.6 (51.8, 59.6)	9.8 (7.9, 12.2)	120.6 (112.1, 129.9)	17.6 (15.3, 20.2)	206.4 (190.5, 223.8)
Single positive	55,985	485	285.0 (260.7, 311.6)	227.2 (199.4, 258.9)	366.7 (324.6, 414.3)	201.9 (179.4, 227.3)	615.3 (537.4, 704.4)
Double positive	14,288	494	1,184.9 (1,084.8, 1,294.3)	936.3 (823.7, 1,064.3)	1,158.8 (1,380.1, 1,760.7)	823.9 (732.5, 926.7)	2,733.4 (2,391.4, 3,124.4)
Any positive	70,273	979	462.1 (434.0, 492.0)	369.6 (337.3, 405.0)	594.5 (545.4, 648.0)	325.8 (299.7, 354.1)	1,012.0 (920.2, 1,112.9)
**Parallel test of risk stratification and FIT**							
Negative	423,031	707	54.9 (51.0, 59.1)	8.3 (6.5, 10.7)	121.1 (112.1, 130.8)	16.2 (13.9, 18.8)	208.7 (191.8, 227.0)
Positive	115,247	1,064	305.6 (287.8, 324.6)	234.8 (214.7, 256.8)	406.2 (374.6, 440.6)	210.8 (194.4, 228.6)	684.9 (626.1, 749.3)
**Parallel test of risk score and FIT**							
Negative	257,256	266	34.2 (30.3, 38.6)	3.3 (2.0, 5.4)	78.6 (69.5, 89.0)	9.6 (7.5 12.4)	133.4 (116.4, 152.9)
Positive	281,022	1,505	175.6 (166.0, 184.7)	105.4 (96.8, 114.8)	274.3 (257.6, 292.0)	101.5 (94.2, 109.4)	465.9 (434.9, 499.2)

**Figure 4:**
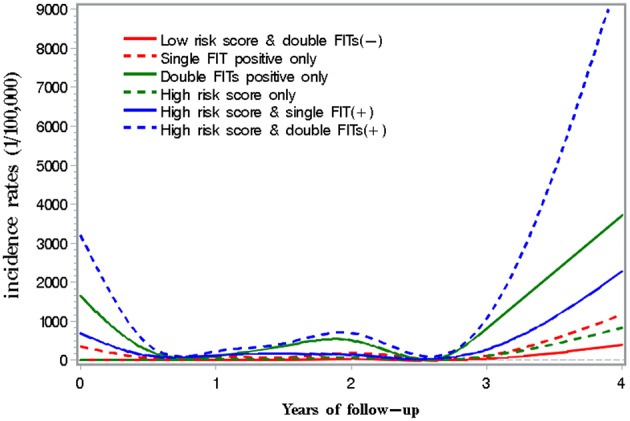
CRC incidence along follow-up time by combined use of FIT with risk score.

Figure 4 presents incidence of CRC along with years of follow-up until December 2017 by results of risk score and FIT. A peak in incidence was observed within 6 months of screening, and then the incidence decreased within 2–3 years of screening. Thereafter, the incidence increased with the follow-up time in each group.

### Validity of Assumed Screening Modalities in Detecting CRC

As presented in Table 5, if all positive subjects received further colonoscopy and diagnostic examinations, the initial screening modality used in the program, i.e., parallel test of FIT and risk stratification, would detect 873 CRC cases, with a sensitivity of 71.4%, specificity of 78.7%, and PPV of 0.76%. One hundred thirty-two colonoscopy examinations were required to detect one CRC case.

**Table 5:** Validity of used and assumed initial screening methods.

**Screening modality**	**No. of subjects**	**No. of CRC**	**Sensitivity (%)**	**Specificity (%)**	**PPV (%)**	**Colonoscopy per detected CRC**	**Sensitivity analysis**^**a**^				
							**No. of CRC**	**Sensitivity (%)**	**Specificity (%)**	**PPV (%)**	**Colonoscopy per detected CRC**
**Risk stratification**											
Low risk	483,014	1,035					1,237				
High risk	55,264	188	15.4	89.7	0.34	294	213	14.7	89.7	0.39	259
**Risk score**											
Risk score < 19	290,464	383					459				
Risk score ≥ 19	247,814	840	68.7	54.0	0.34	295	991	68.3	54.0	0.40	250
Risk score < 24	472,651	883					1,061				
Risk score ≥ 24	65,627	340	27.8	87.8	0.52	193	389	26.8	87.8	0.59	169
**FIT**											
Negative	468,005	404					567				
Single positive	55,985	384	48.7	89.3	0.69	146	421	42.6	89.3	0.75	132
Double positive	14,288	435	51.8	97.1	3.04	33	462	44.9	97.0	3.23	31
Any FIT positive	70,273	819	67.0	86.9	1.17	86	883	60.9	86.9	1.26	80
**Parallel test of risk stratification and FIT**											
Negative	423,031	350					498				
Positive	115,247	873	71.4	78.7	0.76	132	952	65.7	78.6	0.83	121
**Parallel test of risk score and FIT**											
Risk score cutoff point 19											
Negative	257,256	129					184				
Positive	281,022	1,094	89.5	47.8	0.39	257	1,266	87.3	47.8	0.45	222
Risk score cutoff point 24											
Negative	413,631	294					420				
Positive	124,647	929	76.0	77.0	0.75	134	1,030	71.0	77.0	0.83	121

a Sensitivity analysis by defining interval and missed CRC as those diagnosed within 3 years after initial screening tests.

*PPV, positive predictive value*.

We further evaluated validity of assumed risk score-based screening modality. Parallel test of FIT with risk score using the optimal cutoff point of 19 detected more CRC cases than parallel tests of FIT with risk stratification, but at the cost of decreased PPV (0.39%) and doubled colonoscopy examinations for each detected CRC. When using 24 as the cutoff point of risk score, parallel test of FIT with risk score was expected to avoid 56 interval CRCs with a minimal decrease in PPV and an increase in colonoscopy per detected CRC.

## Discussion

In this CRC mass screening program provided by the Chinese government as a major public health service (17), the main findings include the following: (1) risk assessment was complementary to FIT in identifying CRC cases, supporting parallel test of the two methods in the population; (2) the compliance rate was as low as 23.5% in positive subjects, indicating the urgency to optimize initial screening modality in the population; (3) risk score system developed in this study performed better in detecting CRC than risk stratification used in the program, indicating potential benefits by using risk score; and (4) parallel use of FIT and risk assessment performed well in predicting long-term risk of CRC, suggesting that subjects positive in initial screening should be followed up extensively even if they are negative in colonoscopy examinations.

Selection of CRC screening modality depends not only on validity of the modality in target population but also on feasibility, affordability, compliance, and clinical capacity of screening, particularly in resource-limited settings (5). In Shanghai CRC screening program, FIT, the most widely used qualitative CRC screening method, was used to identify high-risk individuals using a cutoff value of fecal hemoglobin (Hb) ≥100 ng/ml (20 μg Hb/g feces) based on evidence from Chinese (18) and other populations (4, 19, 20). In a meta-analysis including 17 studies, the median fecal Hb positivity cutoff was found to be 20 μg Hb/g feces, with a range of 10–200 μg Hb/g feces (21). The detection threshold resulted in high specificity but low sensitivity in our population and thus a large number of interval CRCs, which are usually considered as a failure of detection due to the lack of diagnostic tools with perfect sensitivity and specificity (22).

Combined use of risk stratification and FIT has been performed to achieve higher accuracy than FIT only (23). The importance of risk assessment in initial screening was also supported by Steele et al. (24), who found that interval CRCs were less likely to bleed. Considering that FIT can detect bleeding lesions while questionnaire-based risk assessment helps to identify individuals with lesions not bleeding (25), parallel test of the two methods was developed in 2006 in China as an initial screening modality to improve sensitivity of CRC screening (9) and recommended to the whole country (8). The observed low consistency of risk factors with FIT, as well as the greatly improved sensitivity, strongly supports parallel test of risk assessment and FIT in the population.

In this study, we developed a risk score system based on long-standing risk factors like age, sex, history of any cancer, and family history of CRC that perform well in long-term risk prediction (26), and specific intestinal symptoms such as diarrhea, constipation, mucus bloody stool, and intestinal polyps that had better short-term predictive values for CRC (27, 28). The risk score system was superior to currently used risk stratification in detecting malignant and precancerous lesions and in predicting long-term risk of CRC, but at the cost of almost doubled colonoscopy per detected CRC. It is of note that sensitivity of qualitative FIT was much lower in this study than in a previous report (7). Therefore, the parallel test screening modality should be optimized to trade off validity, compliance to colonoscopy, and clinical capacity of screening by adjusting cutoff point for risk score and by improving stool-based test.

In this study, only 23.5% positive subjects had colonoscopy, lower than 39.8% in the whole population of Shanghai (11). In addition to subpopulation disparity, compliance to colonoscopy in this study may have been underestimated due to the lack of information beyond the 13 designated hospitals. Nevertheless, low compliance to colonoscopy is common around the world, regardless of age, sex, and ethnicity (29), making a large number of missed cases a bigger challenge than interval cases. Validity of screening modality, particularly specificity, has been associated with compliance to colonoscopy (30). Lower specificity of the risk score-based screening modality may further decrease the compliance. Given the low compliance to colonoscopy, the numbers of detected neoplasms in each category of the new risk score strategy may be greatly underestimated. In this study, compliance to colonoscopy was 16.9% among high-risk individuals defined by risk stratification, triple of 5.6% in subjects with high-risk score, indicating potential benefits of using risk score even at the current level of compliance. When we improved specificity of risk score at same level of risk stratification by increasing its cutoff point to 24, we found that the risk score-based screening modality may detect additional 56 CRCs at the cost of additional 9,400 colonoscopy examinations, supporting utility of the risk score system. Moreover, medical insurance, lower educational attainment, discomfort during colonoscopy, fear of complications, and lack of information on colonoscopy procedures were also barriers to colonoscopy screening (31–33), and should be overcome to increase compliance to colonoscopy.

There are several strengths of this study. First, the large sample size makes it possible to evaluate performance of multiple assumed screening modalities. Second, the risk score system was developed with a comprehensive range of risk variables such as age, sex, history of cancers, and intestinal symptoms. All the information are easy to collect (26), ensuring feasibility of the system in the “real world.” Moreover, the record linkage with the Cancer Registry and the Vital Statistics enabled us to collect all CRC cases and to calculate person-years of observations accurately, through which we found that the incidence of CRC decreased sharply after an incidence peak and began to increase between 2 and 3 years after screening, supporting the use of the period to define interval CRC and missed CRC (20, 24, 34). Finally, sensitivity analysis was conducted by defining interval or missed cases as linked CRC diagnosed within 3 years after initial screening. Similar results provide further evidence for our conclusions.

Several limitations should be considered. First, we did not collect information on lifestyle factors such as smoking, alcohol use, red meat intake, and physical activities, which have been included in multiple risk score systems (26, 35). It is possible that these unmeasured confounders may have biased the associations of collected risk factors with the risk of CRC and thus the weighing of each factor in the system. We could not compare the risk score system developed in this study with others due to the lack of lifestyle information to calculate risk score within other systems. Second, we may have underestimated the incidence of CRC in this population because of the lagging in cancer registry. Furthermore, the screening value of risk score system developed in this study was just validated internally. External validation study is needed to verify the extrapolation and generalization of the system. Finally, the follow-up time was not long enough to observe long-term predictive value of the risk score system, in which a longer follow-up is warranted.

## Conclusions

In conclusion, quantitative risk score-based modality may help to improve effectiveness of CRC screening and has potential of scaling up in the population. Cutoff points of risk score should be optimized and stool-based test should be improved for large-scale usage in Chinese population. The effect of the parallel screening modality on improving compliance to colonoscopy and early detection of CRC, as well as its cost-effectiveness in view of society, warrant further evaluations.

## Data Availability

The datasets generated for this study are available on request to the corresponding author.

## Ethics Statement

This study was approved by the Medical Ethics Committee of the Center for Disease Control and Prevention in Pudong New Area of Shanghai, and oral consent was obtained from each participant of the screening program.

## Author Contributions

WW and YW drafted the manuscript. TL and WX conceived and designed the study. CY and BY made substantial contributions to the study design. CY and YZ were responsible for study coordination. YW and BY contributed to data quality control. HJ and XL contributed to data analysis. All authors contributed to the revision of the manuscript and approved the final manuscript.

### Conflict of Interest Statement

The authors declare that the research was conducted in the absence of any commercial or financial relationships that could be construed as a potential conflict of interest.

## Abbreviations

AUC: area under receiver operating characteristic curve
CI: confidence interval
CRC: colorectal cancer
FIT: fecal immunochemical test
PPV: positive predictive value
ROC: receiver operating characteristic curve.
